# Chimeric Antigen Receptor Cell Therapy: Empowering Treatment Strategies for Solid Tumors

**DOI:** 10.3390/cimb47020090

**Published:** 2025-01-31

**Authors:** Tang-Her Jaing, Yi-Wen Hsiao, Yi-Lun Wang

**Affiliations:** 1 Division of Hematology and Oncology, Department of Pediatrics, Chang Gung Memorial Hospital, 5 Fu-Shin Street, Kwei-Shan, Taoyuan 33315, Taiwan; g987669@gmail.com; 2 Division of Nursing, Chang Gung Memorial Hospital, 5 Fu-Shin Street, Kwei-Shan, Taoyuan 33315, Taiwan; apple80168@cgmh.org.tw

**Keywords:** solid tumor, chimeric antigen receptor, natural killer, invariant natural killer T, macrophage, tumor microenvironment

## Abstract

Chimeric antigen receptor-T (CAR-T) cell therapy has demonstrated impressive efficacy in the treatment of blood cancers; however, its effectiveness against solid tumors has been significantly limited. The differences arise from a range of difficulties linked to solid tumors, including an unfriendly tumor microenvironment, variability within the tumors, and barriers to CAR-T cell infiltration and longevity at the tumor location. Research shows that the reasons for the decreased effectiveness of CAR-T cells in treating solid tumors are not well understood, highlighting the ongoing need for strategies to address these challenges. Current strategies frequently incorporate combinatorial therapies designed to boost CAR-T cell functionality and enhance their capacity to effectively target solid tumors. However, these strategies remain in the testing phase and necessitate additional validation to assess their potential benefits. CAR-NK (natural killer), CAR-iNKT (invariant natural killer T), and CAR-M (macrophage) cell therapies are emerging as promising strategies for the treatment of solid tumors. Recent studies highlight the construction and optimization of CAR-NK cells, emphasizing their potential to overcome the unique challenges posed by the solid tumor microenvironment, such as hypoxia and metabolic barriers. This review focuses on CAR cell therapy in the treatment of solid tumors.

## 1. Introduction

Immunotherapy is a powerful cancer treatment, and adoptive cell therapy (ACT) is proving to be particularly effective. Chimeric antigen receptor (CAR)-T cells feature in six FDA-approved treatments for hematological malignancies [1]. CAR-T cell therapy encounters significant hurdles, including elevated production expenses, cytokine release syndrome, immune effector cell-associated neurotoxicity syndrome, and graft-versus-host disease (GVHD) [2,3]. The tumor microenvironment (TME) plays a crucial role in influencing cancer progression and reducing the efficacy of CAR-T cells [4].

To address these limitations and expand therapeutic options, researchers have developed alternative CAR-based therapies. CAR-NK (natural killer) and CAR-iNKT (invariant natural killer T) cell therapies have emerged as promising approaches for treating solid tumors [5]. Commonly used sources of functional NK cells for CAR-NK production include the NK-92 cell line, adult peripheral blood, umbilical cord blood, and induced pluripotent stem cells [6]. CAR-NK cells have shown efficacy in treating breast, ovarian, and prostate cancer [7,8]. CAR-iNKT cells, found in both blood and tissues, show superior antitumor activity in vivo compared to CAR-T cells, especially against tumors not easily accessible through circulation [9]. Both CAR-NK and CAR-iNKT cells show comparable antitumor effects in laboratory settings, but CAR-iNKT cells provide a more robust response in live models [10]. These therapies represent significant advancements in cancer immunotherapy.

CAR-macrophages (CAR-M) are anticipated to improve solid tumor treatment efficacy as a new type of immunotherapy, potentially addressing significant challenges linked to CAR-T/NK therapy [11]. Research indicates that CAR-M cells not only exhibit potent anti-tumor capabilities but also open new avenues for immunotherapy, positioning them as a novel therapeutic strategy against solid cancers [12,13]. This review highlights the differences among CAR-T, CAR-NK, CAR-iNKT, and CAR-M cells, exploring their unique advantages and challenges in cancer treatment. By examining these diverse CAR-based approaches, we aim to provide a comprehensive overview of the evolving landscape of cellular immunotherapies and their potential to revolutionize cancer treatment strategies.

This review seeks to clarify the differences between the four CAR cell therapies, exploring their unique advantages and challenges within the realm of cancer treatment.

## 2. What Can Go Wrong with CAR-T Cell Therapy for Solid Tumors?

### 2.1. Physical Barriers Within the Tumor Microenvironment

CAR-T cell therapy encounters considerable obstacles in the context of solid tumors, largely stemming from the diverse physical barriers present within the TME [4,14]. The TME is a multifaceted ecosystem encompassing cancer cells, stromal tissue, the extracellular matrix, and immune cells, and significantly influences cancer progression and treatment outcomes. The obstacles consist of poorly regulated tumor blood vessels and a thick fibrogenic extracellular matrix, which collectively hinder the effective infiltration and migration of CAR-T cells into the tumor tissue [15]. The architecture of the tumor stroma presents a complex obstacle, as it can create a hostile TME that impedes CAR-T cell penetration [16]. Additionally, factors such as high interstitial fluid pressure further complicate the ability of these engineered T cells to reach and effectively engage with tumor cells [17,18]. Consequently, understanding and addressing these physical barriers is crucial for enhancing the efficacy of CAR-T cell therapies in treating solid tumors.

Overall, overcoming these challenges is essential for improving patient outcomes and expanding the applicability of CAR-T cell therapies beyond hematological malignancies to solid tumors.

### 2.2. Trafficking and Penetration into Neoplastic Tissue

CAR-T cell treatment is constrained in solid tumors because of the absence of chemokines and the presence of a thick fibrotic matrix in the tumor tissue [19]. The recent literature emphasizes that the dysregulated tumor vasculature significantly restricts CAR-T cell access to solid tumors, as it hinders the proper circulation and distribution of therapeutic agents [20]. Localized injection of CAR-T cells is more efficacious in confined tumor locations, with intracranial transport being safe and effective in glioblastoma [21,22]. A comprehensive grasp of the mechanisms that enhance or restrict T cell entry into tumors will influence the potential to enhance CAR-T cell movement [23]. CAR-T cells can be engineered to express receptors that are specific to chemokines, including C-C motif chemokine receptor 2 (CCR2) and CCR4, which support efficient contact with tumor cells [24,25]. Recent research suggests that a promising method to improve CAR-T cell therapy may be through promoting tumor chemokine release, which is more acceptable than traditional methods that target specific tumor chemokine profiles [22]. Researchers are improving the effectiveness of CAR-T cell therapy for solid tumors by manipulating the TME using chemokine signals. A key strategy is to engineer CAR-T cells to overexpress specific chemokine receptors, enhancing their trafficking to the TME and improving the infiltration and targeting of tumor cells [4]. Scientists aim to harness the chemokine system to direct CAR-T cells more effectively into tumors, increasing the likelihood of successful cytolysis of cancer cells [26].

### 2.3. Immunosuppressive Tumor Microenvironment

The TME significantly contributes to immunosuppression via mechanisms including nutrient deprivation and the buildup of toxic metabolites [27]. Modifications in nutrients and signals within the TME can substantially hinder immune responses, resulting in metabolic immune suppression. Strategies to enhance CAR-T cell therapy include reshaping the TME to promote better infiltration and reduce immunosuppressive responses [28]. Studies demonstrate that cancer cells can establish an immunosuppressive metabolic microenvironment by limiting immune cells‘ access to crucial metabolites, thereby impairing their function and facilitating tumor survival [29,30,31]. Moreover, a hostile TME marked by nutrient deficiency and the accumulation of detrimental metabolites can further undermine the efficacy of anti-tumor immunity [27]. Research shows that immune-suppressive mechanisms like hypoxia and inhibitory metabolites cause T cell exhaustion and senescence, complicating CAR-T cell activity. Strategies to enhance function include the targeting of exhaustion pathways [32]. Tumor cells use metabolic reprogramming to evade immune system attacks, altering the TME to modulate immune cell functions. These metabolic alterations affect the function and differentiation of non-tumor cells, inhibiting effector T cell activity and expanding regulatory T cells (Tregs). This imbalance in cytokine and chemokine secretion further enhances the immunosuppressive landscape [33]. Understanding the metabolic dynamics within the TME is essential for developing strategies to mitigate its immunosuppressive effects.

### 2.4. Tumor-Infiltrating Immune Cells Reversed the Hostile Tumor Immune Environment

The presence of myeloid-derived suppressor cells (MDSCs) in the TME poses a significant challenge, as these cells exert potent immunosuppressive effects that impair CAR-T cell functionality [34]. These cells are produced abnormally and recruited to the TME, where they play a significant role in creating an immunosuppressive environment that obstructs effective anti-tumor immune responses [35]. MDSCs inhibit T cell trafficking to tumors and directly suppress CAR-T cell activity through multiple mechanisms, including the production of immunosuppressive cytokines and reactive species [36,37].

Tumor-associated macrophages (TAMs), especially the M2 subtype, contribute to the immunosuppressive environment, complicating CAR-T cell responses [38,39,40]. Ongoing efforts are aimed at addressing MDSCs and TAMs to enhance the efficacy of CAR-T therapies, particularly in the context of hematological malignancies and solid tumors [41,42]. Addressing the immunosuppressive roles of MDSCs and TAMs is essential for enhancing the efficacy of CAR-T cell therapies in oncology.

### 2.5. Soluble Inhibitors Impair the Functionality of CAR-T Cells

Soluble inhibitors, such as B-cell maturation antigen (BCMA) and vascular endothelial growth factor A (VEGF-A), can impair the functionality of CAR-T cells, which are designed to target and eliminate cancer cells [43,44]. These inhibitors disrupt the binding process necessary for CAR-T cell activation and action against tumors. Other soluble factors like prostaglandin E2 (PGE2) and VEGFR-2 also contribute to the dysfunction of CAR-T cells in the TME, making CAR-T therapies in solid tumors challenging [45,46]. These inhibitors limit therapeutic efficacy and hinder clinical application, highlighting the need for strategies to counteract immune suppression.

### 2.6. Immune Checkpoint Overexpression Hinders the Effector Functions of CAR-T Cells

**Figure 1:**
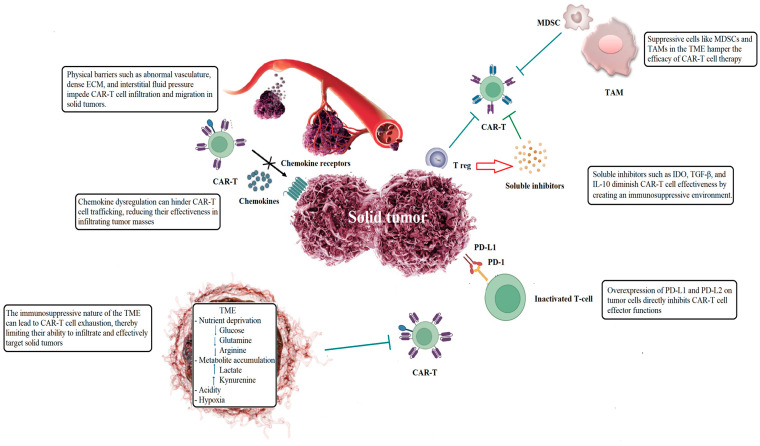
CAR-T cell therapy encounters a variety of obstacles, such as tumor heterogeneity, antigen escape, cell exhaustion, on-target/off-tumor effects, and CAR-T cell-related toxicities including cytokine-release syndrome and neurotoxicity. The most significant obstacle remains the immunosuppressive tumor microenvironment. Tregs, TAMs, MDSCs, and stromal cells contribute significantly to the TME by suppressing immune responses. T cells redirected toward universal cytokine killing release interleukins to boost CAR-T cell activity and longevity while reactivating host immunity. Abbreviations: ECM, extracellular matrix; IDO, indoleamine 2,3-dioxygenase; MDSC, myeloid-derived suppressor cell; PD-L, programmed cell death ligand; TAM, tumor-associated macrophages; TGF-β, transforming growth factor–β; TME, tumor microenvironment; Treg, regulatory T cell.

Research on checkpoint inhibition is crucial for improving CAR-T cell therapy for solid tumors. The overexpression of immune checkpoints like PD-L1 and PD-L2 can hinder the effectiveness of CAR-T cells, making it difficult to achieve desired outcomes [47,48]. Combining CAR-T cell therapy with immune checkpoint inhibitors (ICIs) may improve efficacy and safety, potentially leading to better clinical responses [49]. This innovative approach aims to leverage the strengths of both therapies: CAR-T cells can target and infiltrate tumors that are otherwise immunologically silent, while ICIs can help overcome tumor-induced immune suppression [47,50]. However, challenges remain, such as the downregulation of target antigens by tumor cells under CAR-T therapies. Additional investigation is essential to enhance these combination therapies and completely unlock their potential in solid tumors. Figure 1 illustrates the limitations of CAR-T cell therapy and the challenges it faces in solid tumors.

## 3. Strategies to Address Challenges in CAR-T Cell Therapy for Solid Tumors

CAR-T cell therapy has demonstrated potential in treating hematological malignancies, yet it encounters considerable obstacles in the context of solid tumors. Researchers are exploring strategies to enhance the effectiveness of CAR-T cells in this context. One approach involves optimizing CAR structures by incorporating novel costimulatory domains such as CD28 and 4-1BB, which are crucial for enhancing T cell persistence and antitumor activity [51].

Additionally, addressing the difficulty of CAR-T cell trafficking to tumor sites is crucial, as this limits the cells’ efficacy in solid tumors. Strategies to improve this include modifying T cells with specific cytokine genes and receptors to enhance their functionality within the TME [52].

Moreover, clinical challenges such as cytokine release syndrome (CRS) and the high rates of tumor heterogeneity further complicate treatment outcomes. Recent reviews have summarized these challenges and outlined promising strategies, emphasizing the need for innovative combinations and the evolution of CAR-T cell therapy to overcome these barriers [53]. In summary, ongoing research is focused on refining CAR-T cell therapies to improve their application in solid tumors, addressing both biological and clinical hurdles.

## 4. Overview of CAR-T Cell Therapy Application in Solid Tumors

CAR-T cell therapy has revolutionized blood cancer treatment, but its application in solid tumors faces challenges, resulting in limited effectiveness and inconsistent outcomes in real-world situations. The disparity between clinical trial results and real-world outcomes underscores the complexity of CAR-T cell therapy for treating solid tumors. Second and third generations of CAR-T cell therapy mark advancements in solid tumor treatment. Second-generation cells incorporate co-stimulatory domains like CD28 or 4-1BB, enhancing T cell activation and persistence in the fight against cancer cells. Third-generation cells combine multiple domains, which may enhance the anti-tumor response. These advancements aim to overcome limitations in solid tumors [54].

### 4.1. Clinical Insights on CAR-T Cell Applications for Solid and Brain Tumors

For more than a decade, clinical studies have evaluated CAR-T cells targeting various tumor antigens. Initial research concentrated on first-generation CARs targeting carbonic anhydrase IX (CAIX), CD171, FRα, GD2, or IL-13Rα [55]. However, first-generation CAR-T cells showed limited antitumor activity, except for GD2-CAR/EBV-specific T cells, which responded fully in 3 out of 11 patients. GD2-CAR T cells can produce anti-tumor responses in patients with active neuroblastoma. The persistence of these CAR-T cells at low levels over time is associated with a longer survival time [56]. The induction of cholangitis by CAIX-CAR T cells was expected, given that CARs lacking co-stimulatory endodomains were integrated into T cells and patients did not undergo lymphodepleting chemotherapy before adoptive transfer [57].

A meta-analysis of 22 trials conducted by Hou et al. revealed that CAR-T cell therapy demonstrates the highest efficacy for neuroblastoma while showing minimal effectiveness for gastrointestinal cancers. Furthermore, the effectiveness of the treatment was not notably influenced by various aspects of treatment strategies, including lymphodepletion before T cell infusion, transfection methods, the duration of cell culture, the persistence of CAR-T cells, transfection efficacy, total cell dose, and the administration of IL-2. The length of T cell culture was the only factor associated with enhanced clinical outcomes [58]. The pooled response rate stood at 9%, and treatment success showed no significant variation due to factors such as lymphodepletion, transfection method, or the duration of cell culture. Researchers continue to express optimism regarding future efficacy through additional structural modifications.

### 4.2. Challenges of CAR-T Cell Immunotherapy for Solid Tumors

The design of CARs is modular, comprising an antigen-binding domain, a hinge, and a transmembrane domain, along with an intracellular signaling domain. CAR-T cell therapy is a promising cancer treatment that targets specific antigens on tumor cells, enabling the identification of cell surface proteins without depending on the major histocompatibility complex (MHC). However, the effectiveness of CAR-T therapy depends on the presence of specific human leukocyte antigen (HLA) types, limiting its application to a restricted patient population [59]. CAR-T cells exhibit sensitivity to reduced HLA expression and flaws in the antigen processing pathway, tactics employed by tumor cells to escape immune responses [57]. Initial iterations of CARs featured solely a T cell activation domain; however, subsequent designs have incorporated signaling domains from co-stimulatory molecules [60]. CARs are classified as either second- or third-generation based on the quantity of co-stimulatory molecules present [61].

Despite these challenges, understanding real-world experiences is crucial in optimizing CAR-T cell therapy for solid tumors. Tumor heterogeneity and immune evasion are crucial concepts in cancer biology and treatment resistance. Tumor heterogeneity refers to the diverse characteristics of cancer cells within a single tumor, influencing their interactions with the immune system. Cellular plasticity, particularly dedifferentiation, helps tumors to evade detection [33]. Table 1 presents the counterstrategies for the challenges associated with CAR-T cell therapy. Further exploration and innovation are needed to enhance its effectiveness in this area.

## 5. If CAR-T Therapy Is Unsuccessful, Should We Consider an Alternative Approach?

CAR-T cell therapy offers a promising option for cancer treatment, but its effectiveness can be hindered by factors like tumor heterogeneity, antigen escape, and cell exhaustion. T cell exhaustion or activation-induced CAR-T death has been suspected to contribute to the poor persistence of CAR-T cells [4]. In cases where CAR-T fails, alternative strategies like a second dose and genetic modifications may improve its efficacy [74,75,76]. Current CAR-T therapies have limitations, particularly in specific cancers like leukemia and pancreatic cancer, indicating that alternative approaches may be needed when first-line treatments fail to produce results [77]. Some researchers support the development of next-generation CARs or other immunotherapies targeting various tumor antigens, potentially opening new treatment avenues [78]. This highlights the importance of tailored approaches in oncology.

### 5.1. CAR Race Towards Cancer Immunotherapy: Exploring CAR-NK, CAR-iNKT, and CAR-M Therapies

**Figure 2:**
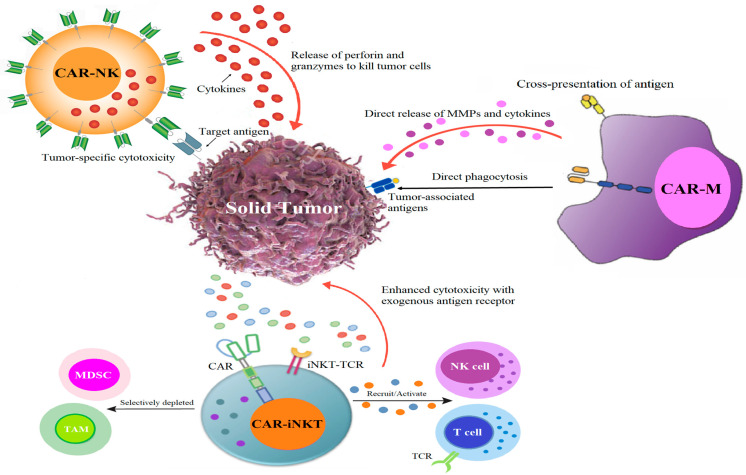
An overview of the mechanisms through which CAR cells derived from various cell types eliminate tumor cells. CAR-NK cells are engineered to specifically seek out and eliminate tumor cells by utilizing cytotoxic mechanisms. They release cytolytic granules that contain perforin and granzymes, leading to cell lysis and apoptosis. CAR-iNKT cells can directly induce cytotoxicity against tumor cells without relying on MHC interactions, enhance the immune response, and migrate to tumor sites through the expression of chemokines. Most importantly, CAR-iNKT could recognize and inhibit CD1d-positive TAMs and MDSC, which promote tumor progression and protect tumors from the attack of immune effectors through buildup of an immune-suppressive environment [81]. Their unique ability enhances their effectiveness in targeting tumors through CAR-dependent and CAR-independent cytotoxicity. CAR-Ms use macrophages’ unique properties to enhance anti-tumor responses. They bind to tumor-associated antigens, trigger phagocytosis, and stimulate pro-inflammatory cytokines, thereby creating a robust immune response against cancer cells. Abbreviations: MDSC, myeloid-derived suppressor cells; TAM, tumor-associated macrophages.

When CAR-T therapy fails, the exploration of alternative options like CAR-NK, CAR-iNKT, or CAR-M therapies becomes increasingly relevant in the landscape of cancer treatment. CAR-NK cells retain natural cytotoxicity, allowing them to target tumors even when cancer cells downregulate antigen expression [78]. CAR-iNKT cells combine natural killer T cells with CAR technology, enhancing effectiveness against various tumors while minimizing toxicity [79]. CAR-M cells, derived from macrophages, penetrate tumors more effectively and exhibit enhanced antitumor efficacy with reduced toxicity [80]. These therapies offer distinct advantages for personalized cancer immunotherapy. Figure 2 illustrates the killing mechanisms of CAR-NK, CAR-iNKT, and CAR-M cells.

CAR-NK cells present numerous benefits when contrasted with CAR-T cells. Production can occur using established cell lines or allogeneic NK cells that lack matched MHC [82]. Furthermore, they possess the ability to eradicate cancer cells through both CAR-dependent and CAR-independent pathways, while demonstrating diminished toxicity, especially regarding cytokine release syndrome and neurotoxicity [83,84]. A phase I clinical trial is presently in progress, concentrating on individuals with relapsed/refractory mantle cell lymphoma, to assess the safety and tolerability of this treatment [85]. Macrophages infiltrate tumors adeptly, act as crucial immune regulators, and are plentiful within the tumor microenvironment. M2 immunosuppressive macrophages demonstrate effectiveness comparable to pro-inflammatory M1 macrophages in the phagocytosis of target cells, and they possess the capability to be induced into the M1 phenotype [38,86]. There is significant enthusiasm surrounding the advancement of CAR macrophages for cancer immunotherapy, aimed at tackling critical challenges associated with CAR T/NK therapy, especially in the context of solid tumors [80]. Both CAR-NK and CAR-M present unique challenges. This review article examines the present landscape and significant challenges for CAR-T and CAR-NK therapies, subsequently addressing the structure and recent advancements in the development of CAR-M as cancer-specific phagocytes, antigen presenters, immunostimulators, and TME modifiers.

### 5.2. CAR-NK: An Encouraging Substitute for CAR-T Therapy

NK cells act as the primary line of defense within the innate immune system, focusing on the identification and destruction of virus-infected and cancerous cells. NK cells are not dependent on HLA matching, like T cells are, which positions “off-the-shelf” NK cell therapy as a practical alternative [87]. *Similar to* CAR-T cells, the precise interaction of CAR with its target antigen on tumor cells triggers CAR-NK cells, resulting in the release of perforin and granzymes that eliminate tumor cells. At present, the majority of CAR-T therapies employ autologous T cells, whereas CAR-NK cell therapies can originate from allogeneic donors [88].

CAR-NK employs a range of techniques for gene transduction, such as retroviruses, lentiviruses, electroporation, liposomes, and DNA transposons [89,90]. These approaches present drawbacks, such as possible cancer risks and the inhibition of primary NK cell viability. Lentiviral systems are efficient for genome engineering, particularly in gene therapy and CRISPR/Cas systems. They efficiently deliver large, complex transgenes, positioning them as optimal candidates for stable gene transfer in disease treatment [91]. Electroporation and liposome transfection facilitate the rapid introduction of exogenous genes; however, they do not integrate these genes into the genome of the target cell [92]. DNA transposons present benefits such as minimal immunogenicity, enhanced biosafety, and reduced production costs [93]. However, they need to overcome low efficiency and NK cell death. CAR-NK’s application in solid tumors requires a design that bypasses or improves the TME [94].

CAR-NK therapy is a unique approach to cancer treatment involving the replacement of carrier cells with NK cells. NK cells exhibit cytotoxic capabilities and operate in a manner akin to CD8+ T cells. These can originate from multiple sources and possess the ability to eliminate tumor cells and cells infected by viruses without the need for prior sensitization [95]. The design of CAR-NK cells considers several critical factors, including autocrine cytokines, the metabolic composition of tumors, and the tumors’ capacity to evade immune responses. This treatment is promising due to its unique processes and adaptability to various TMEs. It eradicates cancer cells through direct cytotoxicity, cytokine release, and antibody-dependent pathways. The safety profile of CAR-NK therapies renders them a feasible alternative to conventional treatments, targeting tumor heterogeneity and inhibiting immune responses [96].

Hypoxia represents a pathological process that leads to abnormal alterations in tissue metabolism, function, and structure, stemming from inadequate oxygen supply or issues in oxygen utilization. This can encourage the development of new blood vessels, and also modify energy metabolism, support immune evasion, trigger invasion and metastasis, initiate pro-tumor inflammation, sustain proliferative signals, and lead to genomic instability. Hypoxia results in the increased expression of hypoxia-inducible factors and decreased phosphorylation levels of extracellular signal-regulated kinases and signal transducer and activator of transcription 3 (STAT3), along with diminished cytotoxicity of NK cells [97]. It also increases autophagy levels and enhances the degradation of granzyme B.

The hypoxic microenvironment in tumor cells accumulates adenosine triphosphate, inhibiting NK cell maturation and promoting tumor metastasis, with CD73 expression upregulated during breast cancer and sarcoma growth [98]. Hypoxic conditions inhibit the nuclear factor of activated T cell production, increase MDSCs, and decrease NK cell activity [99]. Research indicates that high lactate levels associated with hypoxia can inhibit the production of interferon-gamma (IFN-γ) by T cells, leading to reduced NK cell activity due to the inactivation of critical transcription factors like hypoxia-inducible factors-1α [100].

### 5.3. CAR-iNKT Immunotherapy: A Novel Path for CAR-Based Cancer Immunotherapy

Researchers are using CAR technology to enhance iNKT cells’ tumor-targeting capabilities, aiming to overcome barriers in solid tumor therapy. These iNKT cells, characterized by their semi-invariant T cell receptor, bridge innate and adaptive immunity, modulate TME, and exhibit intrinsic resistance to exhaustion. iNKT cells represent a compelling option for CAR-based cancer immunotherapy, given their strong antitumor responses along with their direct killing and adjuvant effects [81]. NKT cells, which encompass iNKT cells, are T-lineage cells that do not rely on MHC restriction and can develop into mature iNKT cells upon recognizing glycolipid antigens. They generate immunomodulatory factors and tumor-eradicating cytokines, supporting adaptive defense and eliminating TAM [101]. Current research on synthetic glycolipid activators seeks to utilize these cells for therapeutic purposes, potentially providing novel strategies for cancer treatment [102].

#### 5.3.1. Development of iNKT Cells

iNKT cells possess a unique T cell receptor (Vα24-Jα18 and Vβ11) that allows them to identify glycolipidic antigens through non-polymorphic class I-like HLA molecules (such as CD1d) [103,104]. They are capable of releasing both Th1 and Th2 cytokines, which enhance adjuvant effects and promote the maturation of NK cells and dendritic cells (DCs). They stimulate IFN-γ-producing iNKT cells and can mediate a robust antitumor response through cytolysis or tumor cell death via the Fas/FasL pathway. IL-12 secreted by DCs also stimulates iNKT cell production [105].

#### 5.3.2. Antitumoral Role of iNKT Cells

iNKT cells, which are restricted by CD1d, can be activated both in vitro and in vivo; however, their frequency remains low because of their limited presence in human peripheral blood mononuclear cells. Combining α-GalCer with DCs enhances the iNKT cell survival rate. However, repeated infusions are required, and combining α-GalCer with TLR9 stimulation improves activation. iNKT cells are divided into three subsets, secreting Th1 and Th2 cytokines, and their regulation remains unclear [106]. CD8+ iNKT cells, recognized for their Th1 cytolytic activity, are effective in targeting tumors, while CD4+ iNKT cells produce Th2 cytokines. These cells regulate immune responses and exert antitumor effects, underscoring their potential in cancer immunotherapy. Understanding these subtypes can lead to more effective therapeutic approaches. Future immunotherapy strategies for iNKT cells should prioritize the enhancement of CD4− iNKT cell production, as this has been acknowledged as a crucial avenue for improving therapeutic outcomes in cancer treatment [81]. The ability of iNKT cells to rapidly respond to pathogens and produce cytokines positions them as essential players in the immune response, underscoring the significance of optimizing their function for effective immunotherapy.

#### 5.3.3. iNKT Protects from GVHD

The various groups of iNKT cells, such as iNKT1, iNKT2, and iNKT17, enhance both proliferation and migration in mice following gene transplantation. They regulate GVHD via IL-4 production and possess immunomodulatory characteristics [107]. GVHD stands as the primary cause of morbidity and mortality in allogeneic hematopoietic cell transplantation, with iNKT cells being presented by CD1d molecules rather than being restricted by MHC [108]. iNKT cell immunotherapy effectively prevents GVHD in mouse models through the inhibition of conventional DCs; however, immune rejection arises when autologous T cells identify non-self peptide MHC [99].

Comprehensive preclinical and clinical evidence underscores the promising role of iNKT in safeguarding against GVHD, highlighting the potential of iNKT-based immunotherapy derived from healthy donors without the associated risk of GVHD [109].

#### 5.3.4. Essential Cytokines Enhance CAR-iNKT Activity

Cytokines play a crucial role in immune effector cell activation, proliferation, differentiation, and immigration, including CAR-based immunotherapy. Recent research highlights the role of STAT5-related cytokines (IL-2 and IL-15) in tumor immunotherapy efficacy, with IL-15 cytokines inhibiting tumor growth and preventing metastasis, making them promising cancer treatment strategies [110]. IL-23 promotes memory T cell proliferation and provides a selective proliferation signal [81,111].

iNKT cells have shown antitumor efficacy in preclinical studies and clinical trials, but limited expansion hinders clinical application. Synthetic activators with specific effects could improve understanding and clinical application. Contemporary high-throughput sequencing methods have the potential to enhance our comprehension of iNKT cell characteristics at the single-cell level [81,101].

### 5.4. CAR-Macrophage: Pioneering Advancements in Cellular Immunotherapy

Macrophages, as immune system components, offer potential for CAR-based therapies due to their ability to penetrate tumors, influence the TME, and maintain anti-tumor responses [112]. CAR-M is a groundbreaking approach in cancer immunotherapy, enhancing anti-tumor responses through the interaction between tumor-associated antigens and CAR receptors. This method integrates CAR technology, providing a more secure and resilient method for cancer immunotherapy [11]. CAR-Ms target antigens for tumor clearance and fibroblast activation protein for liver fibrosis treatment [113]. They use CD3ζ, a signaling domain, and dual-signaling CARs to enhance target phagocytosis and resistance to M2 polarization [112,114]. Macrophages are categorized into M1 (pro-inflammatory) and M2 (anti-inflammatory) subtypes, regulated by key pathways [115].

CAR-Ms from various sources have advantages and limitations, including Tamm–Horsfall Protein-1 (THP-1) cell lines, monocytes, bone-marrow-derived macrophages (BMDMs), and pluripotent stem cells (PSCs). THP-1 offers stable genetics, BMDMs provide raw materials, and PSCs offer ease of amplification [12,116].

#### 5.4.1. Preclinical and Clinical Studies of CAR-Ms

Researchers have developed various antigen receptors, including CAR-P, HER2 CAR, CAR-iMac, injectable gene nanocarrier–hydrogel superstructure, and mosaic antigen receptor-modified macrophages, which have shown potential in cancer immunotherapy, but further research is needed to determine their safety and effectiveness in humans [111].

A clinical trial in the UK has demonstrated that autologous macrophage therapy derived from peripheral blood monocytes is both safe and effective for patients with cirrhosis. The study reported a reduction in severe adverse events and a lower mortality rate within 360 days, representing a significant advancement in this area of research [117].

#### 5.4.2. The Advantages, Obstacles, and Prospective Trajectory of CAR-Ms

CAR-Ms represents an innovative approach to cellular immunotherapy, offering distinct benefits compared to CAR-T and CAR-NK therapies. They can penetrate tumor tissues, diminish antigens, and enhance tumor therapy. They can also reprogram the tumor microenvironment, directly eliminate antigen-expressing cells, and establish a pro-inflammatory environment [112,118]. CAR-M therapy encounters challenges related to time and cost; however, advancements in rapid production and in vivo induction strategies are currently being pursued. Macrophages have the potential to enhance effectiveness, as clinical trials have validated their safety and efficacy [119].

#### 5.4.3. The Future Direction of CAR-M Therapy

Further research is needed to enhance the effectiveness of CAR-M therapy, including through structural optimization, genetic engineering, and safety considerations. Non-viral delivery systems can program macrophages into CAR-Ms, aiding tissue reconstruction and resolving pulmonary alveolar proteinosis [120].

#### 5.4.4. CAR-M Therapy Alongside Additional Immunotherapeutic Approaches

Researchers are exploring the combination of CAR-Ms with other immunotherapies to enhance tumor inhibition, reduce CRS risk, and reduce neurotoxicity, as CAR-Ms and CAR-T cells show synergistic cytotoxicity [98]. CAR-T therapies, when combined with targeted drugs, can enhance tumor infiltration, enhancing their effectiveness against untreatable cancers. This promising pathway also offers potential for CRISPR/Cas9 gene editing [121].

## 6. Conclusions

ACT, especially CAR-T cell therapy, has revolutionized cancer treatment by utilizing the immune system to target and destroy cancer cells. Antigen escape and immune evasion present considerable challenges in CAR-T cell therapy, leading to treatment failure and tumor recurrence. The mechanisms by which antigens escape enable tumor cells to avoid detection and elimination by CAR-T cells, resulting in insufficient therapeutic responses [122,123]. There are ongoing concerns about the development of secondary malignancies after CAR-T cell therapy. This has prompted concerns and demands for thorough studies to gain a clearer understanding of these risks and their long-term effects on patients.

Given these challenges, interest is increasing in alternative cell therapies, including NK cells, iNKT cells, and macrophage cells, which could provide new treatment options. CAR-M cells are emerging as a promising option for solid tumors because of their unique capabilities and effectiveness in targeting TME. Advanced infiltration into tumors enables precise therapy adjustments, potentially enhancing patient outcomes and reducing risks of secondary malignancies. Table 2 provides a comparative analysis of the mechanisms, advantages, and limitations associated with various CAR cell therapies, highlighting the benefits of CAR-T in hematological malignancies and contrasting these with the challenges encountered in solid tumors.

In summary, CAR-T cell therapy has transformed cancer treatment, but the associated risks require further investigation into secondary malignancies and the exploration of alternative therapies such as CAR-M cells to improve treatment efficacy and safety. This review explores the latest advancements in CAR cell therapy and the potential future developments of this immunotherapy treatment approach.

## 7. Future Perspectives

Induced PSC-derived CAR-T cells are emerging as a novel strategy in allogeneic cancer immunotherapies, potentially transforming treatment methods by offering more accessible and effective options for patients [124]. The field faces challenges, especially with solid tumors, where CAR-T therapies have shown limited efficacy. Recent studies are tackling these challenges, offering insights into the development of CAR-T cell therapy and the ongoing obstacles [125].

The future of CAR cell therapy looks promising, with research focused on addressing current limitations and broadening its applications across different cancer types. Further exploration of these advancements is expected to influence the future of immunotherapy treatments.

## Figures and Tables

**Table 1:** Strategies and outcomes to mitigate challenges in CAR-T cell therapy.

Current Challenges	Strategies and Outcomes
Ameliorating CAR-T cell trafficking to solid tumors	Enhancing CAR-T cell efficacy by targeting the TME: The TME significantly impacts immune cell function, with major immunosuppressive mechanisms including upregulated checkpoint receptors, soluble suppressive cytokines, altered chemokine expression, aberrant vasculature, complex stromal composition, hypoxia, and abnormal tumor metabolism [62]; Radiation and oncolytic virus intra-tumoral administration were studied [63].
2. Overcoming hypoxic TME	Overcoming hypoxia in the TME is crucial for improving cancer treatment outcomes: Direct oxygen delivery to tumors, in situ oxygen generation, and enhancing intratumoral oxygen levels through various methods [64]; Overcoming hypoxia-mediated tumor progression: combinatorial approaches targeting pH regulation, angiogenesis, and immune dysfunction [65].
3. Counteracting metabolic challenges	Enhanced anti-tumor effects of hypoxic tumor mesenchymal stem cells: RIAD-CAR and BAY 60-6583 are used for enhanced anti-tumor effects [66]; Lactate dehydrogenase blockade explored alongside CAR-T cell immunotherapy [67]; Optimizing CAR-T cell metabolism, including PD-1/PD-L1 axis blockade, GLUT-1 inhibitors, and CRISPR/Cas9 technology, crucial for tumor metastasis treatment [68].
4. Reversing CAR-T cell exhaustion	CAR-T cell exhaustion impairs the efficacy of immunotherapies, leading to reduced persistence and killing activity of these engineered T cells [69]: Immune checkpoint blockade, targeting the PD-1/PD-L1 signaling pathway, has shown improved outcomes when combined with CAR-T cell therapies in various tumor types [70]; The combination of PD-1 blockade and scFv engineering demonstrates promising outcomes [71,72].
5. Overcoming tumor heterogeneity	Innovative engineering strategies to enhance CAR-T cell effectiveness in solid tumors: Targeting multiple tumor-associated antigens; Co-expressing and secreting BiTEs using CAR-T cells; Applying CARs targeting adapter molecules; BiTE-secreting CAR-T cells successfully overcome antigen heterogeneity; Universal CARs use adaptor elements as ligands; Vaccines, including viruses or dendritic cells, activate CAR-T cells in vivo, with nanoparticles or oncolytic viruses modified to carry drugs, genes, or stimulatory cytokines [68].
6. Counteracting immunosuppressive TME	CAR-T cell therapy resistance to TME-induced immunosuppression [73]: Disrupts the function of immunosuppressive cytokines and their associated signaling pathways; Enhances the release of pro-inflammatory cytokines; Depletes immune suppressor cells in the tumor microenvironment; Strategies include blocking inhibitory pathways, releasing mAbs, and targeting CD-47; The risk of grade 3 neurotoxicity and cytokine release syndrome in CAR-T cell therapy for solid tumors varies across studies, with severe neurotoxicity occurring in 21.7% of patients.

Abbreviations: CAR, chimeric antigen receptor; PD-1, programmed cell death protein 1; TME, tumor microenvironment.

**Table 2:** Mechanisms, advantages, and limitations of various CAR cell therapies.

Type of Therapy	Mechanisms	Advantages	Limitations	Study Number (ClincalTrials.gov)
**CAR-T cells**	CARs bind to tumor cell surface antigens, eliciting anti-tumoral effects through inflammatory cytokines, cytolytic effector function, and the activation of the graft-versus-tumor effect through TNF-related apoptosis-inducing ligand binding to death receptors.	Highly effective against hematological malignancies due to their interaction with circulating tumor cells in the bloodstream or bone marrow; Long-term tumor control.	Effectiveness in solid tumors is hindered by off-target effects due to the heterogeneity of solid tumors and the absence of specific tumor antigens; Higher incidence of CRS and neurotoxicity; GVHD effect; Donor heterogeneity.	As of 25 January 2023, there are 1087 CAR-T cell clinical trials listed on ClinicalTrials.gov
**CAR-NK cells**	NK cells have spontaneous cytotoxic activity and can generate target cell death independent of tumor antigen.	Low risk of GVHD; “Off-the-shelf” manufacturing; Less CRS and neurotoxicity.	Short lifespan; Solid tumor infiltration.	NCT03056339 NCT03415100 NCT02944162
**CAR-iNKT cells**	iNKT cells exhibit NK-like cytotoxicity, capable of directly eliminating tumor cells and infected cells through the secretion of granzyme and perforin, as well as via Fas/FasL-mediated pathways.	Superior antitumor efficacy; Low risk of GVHD; Better adaptability to TME; Broader antitumor spectrum.	Scarcity of iNKT in humans; Tumor antigen heterogeneity and immune evasion; Challenges in standardized manufacture; Concerns about safety and immunotoxicity.	NCT03774654
**CAR-M cells**	CAR-Ms effectively destroy cancer cells through tumor-associated antigen-induced phagocytosis, secreting pro-inflammatory cytokines that activate T cells and remodel the TME, bolstering the immune response against tumors.	Potential advantages in homing in on and infiltrating solid tumors.	Unlike T cells, macrophages do not proliferate effectively either in vitro or in vivo after injection, which restricts their therapeutic potential.	NCT04660929

Abbreviations: CAR, chimeric antigen receptor; CRS, cytokine release syndrome; GVHD, graft-versus-host disease; NK, natural killer; iNKT, invariant natural killer T; TME, tumor microenvironment.

## Data Availability

Not applicable.
